# Preoperative Education for Hip and Knee Replacement: Never Stop Learning

**DOI:** 10.1007/s12178-017-9417-4

**Published:** 2017-06-24

**Authors:** Paul K. Edwards, Simon C. Mears, C. Lowry Barnes

**Affiliations:** Department of Orthopaedic Surgery, University of Arkansas for Medical Sciences, 4301 West Markham Street, Slot 531, Little Rock, AR 72205 USA

**Keywords:** Alternative payment model, Hip replacement, Knee replacement, Bundled payment, Discharge disposition, Hospital readmissions

## Abstract

**Purpose of review:**

Participation in alternative payment models has focused efforts to improve outcomes and patient satisfaction while also lowering cost for elective hip and knee replacement. The purpose of this review is to determine if preoperative education classes for elective hip and knee replacement achieve these goals.

**Recent findings:**

Recent literature demonstrates that patients who attend education classes prior to surgery have decreased anxiety, better post-operative pain control, more realistic expectations of surgery, and a better understanding of their surgery. As a result, comprehensive clinical pathways incorporating a preoperative education program for elective hip and knee replacement lead to lower hospital length of stay, higher home discharge, lower readmission, and improved cost.

**Summary:**

In summary, we report convincing evidence that preoperative education classes are an essential element to successful participation in alternative payment models such as the Bundle Payment Care Initiative.

## Introduction

Providing health care by alternative payment models (APM) is intended to improve the patient experience, improve the quality of health, and reduce the per capita cost of health care. Achieving this triple aim has become the core mission of the Institute for Healthcare Improvement (IHI). In 2013, the Center for Medicare and Medicaid Services (CMS) implemented the Bundled Payments for Care Improvement (BPCI) initiative in order to accomplish these goals. Increasing demands for hip and knee replacement in the aging population makes elective hip and knee replacement (DRG 470 & 469) an ideal episode of care for participation in the BPCI.

Bundled payment models seek to align surgeons and hospitals by placing them at risk for financial penalty if adequate outcomes are not achieved or, oppositely, gain sharing opportunity if specified goals are reached. Successful participation depends on minimizing adverse events while simultaneously minimizing the associated costs. Regarding hip and knee replacement, hospital length of stay (LOS), discharge to post-acute care (PAC) facilities, and hospital readmission are the primary drivers of cost during the episode of care [1–5].

Recent data supports the concept that cheaper care can be of higher quality. Shorter hospital LOS during the surgical admission as well as discharge to home rather than an extended nursing facility reduces readmissions and cost [2–6]. However, only recently has much been written regarding the actual steps to successfully accomplish these objectives [7•, 8, 9•, 10•, 11–13]. Many of these recent reports have proposed that preoperative education plays a critical role to achieve these goals [7•, 14–24]. The aim of this paper is to focus on preoperative education and its role in elective hip and knee replacement. The purpose of this review is to provide compelling evidence supporting the use of preoperative education programs for elective hip and knee replacement to safely achieve the triple aims developed by the IHI.

## What Is Preoperative Education?

Cochrane© review defines *preoperative education* as any educational intervention delivered before surgery that aims to improve people’s knowledge, health behaviors, and health outcomes. Interestingly, the 2014 Cochrane© review concluded that preoperative education provided little benefit above standard patient care for hip or knee arthroplasty [25]. However, five of the nine studies used written, audiovisual, or a combination of these methods to educate the patient [14, 16, 20, 26]. Most educators believe a vital component to the program includes a live class with in-person educators to teach the material and more importantly answer any remaining questions [16]. Since evidence has shown a patient’s willingness to proceed with surgery may be influenced by how completely their questions are answered, the live class approach likely contributes to observed improvement in outcomes [27].

Changes in medical care with bundle payment models have also led to changes in patient education needs. Improving health before surgery as well as emphasizing the importance of family support and early discharge home have not been stressed in the past. The content of preoperative education varies, but typical education material contains information related to pre-surgical processes, the actual steps in the surgical procedure, discharge disposition, postoperative care, potential surgical and non-surgical complications, answers to frequently asked questions, postoperative pain management, and important contact numbers or emails. The format of education ranges from one-to-one verbal communication, patient group sessions, and video or booklet.

## Preoperative Education

### Does It Work?

Although not a new concept, there is a growing body of literature reporting improved outcomes when information and education regarding a patient’s upcoming orthopedic surgery is provided in a timely manner [2–31]. Preoperative education classes have reported decreased pre- and post-operative anxiety, decreased post-operative pain, better coping, improved LOS, increased discharge to home, lower readmissions, and improved costs [6, 7•, 14–24, 32]. Preoperative programs have also been shown more beneficial than post-operative education programs [19].

Moulton et al. acknowledged that while numerous studies have demonstrated improvement in patient outcomes following the introduction of rapid recovery programs, the element of preoperative education remains controversial [24]. Yet, recent reports have shown that implementing clinical pathways (CP) *with* preoperative educational programs can successfully minimize LOS, decrease readmissions, and improve home discharge [6, 7•, 11–13]. Although these comprehensive CP programs are multi-faceted, the educational component is most critical for success. Recent data showed that preoperative education as a single intervention decreased LOS following total knee arthroplasty with no increase in complications or readmissions within 90 days of discharge [33].

### Expectations

Individuals undergoing joint replacement surgery have high expectations for their outcomes [34–38]. A strong correlation has been reported between patient satisfaction and fulfillment of pain relief and functional outcome [39]. For example, evidence shows that up to 20% of all total knee arthroplasty patients are not satisfied with their outcome and the strongest predictor was not meeting their expectations [34, 40, 41]. Preoperative education may improve patient expectations prior to surgery [38]. Patient expectations for pain relief and functional outcome have also been shown to be higher than their surgeons’ expectations [42]. Aligning patient and surgeon expectations preoperative may lead to improved patient satisfaction after joint replacement. Education programs provide an opportunity to further define the surgeon and patient expectations before the elective surgery.

Preoperative education may also prepare patients psychologically for rehabilitation aims by providing them with clear expectations of the recovery process [43]. Providing the patient with adequate information may also increase their sense of responsibility for a successful surgery as well as improve the belief that they are able to cope with the surgery [12, 44].

### Anxiety

Pain after total knee replacement correlates with increased preoperative anxiety levels [45]. Preoperative education may decrease patient anxiety related to an upcoming surgical procedure. Studies have reported that reducing preoperative anxiety in patients improves postoperative recovery, leads to higher levels of patient satisfaction with their surgical experience, and reduces levels of self-reported pain up to 1 year after surgery [16, 45, 46]. A recent non-experimental, descriptive study reported that 78% of participants believed that preoperative education reduced their anxiety prior to elective orthopedic surgery [47]. Several studies have evaluated the most effective means to improve patient anxiety prior to surgery and determined that providing information regarding the upcoming surgery and subsequent hospitalization is most beneficial [17, 18, 48–52] .

The continued existence of racial, ethnic, and gender disparities in total hip and knee replacement are well documented [53–56]. Several studies report that African-Americans had greater fear before and after joint replacement, had less knowledge than whites regarding joint replacement surgery, and were less likely to know someone that had undergone joint replacement [57–60]. For those patients undergoing hip and knee replacement, health beliefs including trust of healthcare providers are also critical issues for certain ethnic groups [59]. Studies also report women ask more questions related to their upcoming surgery and generally have higher anxiety levels regarding their surgery [48, 61]. Preoperative education classes provide another opportunity to distribute additional educational information that may assuage some of the fears in those patients with higher anxiety.

### Health Literacy

Health literacy as defined by the National Network of Libraries of Medicine is the degree to which individuals have the capacity to obtain, process, and understand basic health information and services needed to make appropriate health decisions [62]. Health literacy remains vital in achieving a patients’ understanding of their upcoming surgery and is considered the single best predictor of an individual’s health status [63–65]. However, only 12% of adults have proficient health literacy, according to the National Assessment of Adult Literacy [66]. Those patients with poor health literacy have decreased medical knowledge, poorer health-related outcomes, lower treatment satisfaction, increased hospitalizations, and worse communication with healthcare providers [63–65, 67–69]. For optimal comprehension and compliance, patient education material should be written at a sixth-grade or lower reading level, preferably including pictures and illustrations [70]. Yet, 81% of the patient education materials provided by the American Academy of Orthopaedic Surgeons (AAOS) had a readability score *above* the 8th grade level, which is the mean adult reading level in the USA [71•, 72–75]. Other studies report similar readability of patient education materials across multiple surgical subspecialties including material provided by the American Society of Hand Surgery [76–78].

Providing education materials at the literacy level of our patients will improve their understanding of surgery, minimize anxiety, and improve outcomes that are clinically significant. Providing this material in plain language is vital to improving health literacy. Plain language is communication that users can understand the first time they read or hear it [79]. Patient education material can be more understandable by organizing information in order of importance, use active voice, shortening sentences, using simpler terms, avoiding medical jargon, and adding descriptive pictures [18, 79–81]. Additional formats such as YouTube videos tailored to a 6th grade level, may improve communication and further reduce preoperative anxiety [82, 83]. Focusing efforts to improve the readability of all preoperative education print material will improve the health literacy of orthopedic patients undergoing elective hip and knee replacement.

### Social Support

Previous studies have clearly demonstrated a strong link between patient outcomes and a patient’s social support system. A patient’s social support is associated with mortality, mental health, stress, and depression [84–87]. One study noted that perceived social support was an important factor after hip or knee replacement and that hospital nurses tended to determine the amount of social support by the number of visitors [88]. Preoperative education classes for elective total joint replacement have been shown to promote a sense of social connectedness while also fostering participants’ independence [89]. A recent study by Mitchinson et al. concluded “limited social connectedness impacts negatively on the quality and rate of recovery after major operation, regardless of post-operative complications” [86]. In another study, patients reported that they were less anxious about their surgery as a result of attending preoperative classes, and the preoperative teaching by the multidisciplinary team was effective and highly valued [90]. The authors of this study concluded that a family member or caregiver ideally should be present with the patient during the preoperative education classes in order to better prepare for the upcoming surgery. Many other studies also consider the use of a family member/caregiver referred to as a “Coach” as a critical aspect to successful outcomes after surgery [7•, 8, 91–94]. In addition to requiring patients attend preoperative education class with a designated Coach, we have patients sign non-binding contracts clearly defining family members and friends in their social network that may be available to provide care during the acute surgical time period [8].

Patients with a strong social support have shorter hospital stays, are more likely to be discharged home, more likely to meet ambulation and transfer-out-of-bed targets, score hospital quality of care higher, and are more confident and ready to go home on discharge [91]. Three presence intervals were also found to be significant predictors of key outcome measures: family/friend presence during the preoperative classes, family/friend presence in the preoperative holding area, and family/friend presence during the last physical therapy session [91]. Importantly, education classes allow our healthcare team members to identify and educate these important crucial members of the patient’s social network. Rarely, a patient has no social support. In these circumstances, the problem can be identified in class and the need for a coach can be stressed or discharge plans can be altered.

### Unforeseen Barriers to Home Discharge

Unforeseen barriers to home discharge can delay discharge if not identified and addressed *prior* to surgery. In our experience, the education class often identifies recent changes in the patient’s social family support system, an inability to obtain needed durable medical equipment, failed arrangements for transportation to outpatient physical therapy, unrealistic expectation of discharge to rehabilitation hospital, and/or other issues that may hinder timely discharge. Live education classes with in-person healthcare providers allow each of these concerns to be identified and addressed before surgery, and if not resolved, surgery is delayed until a solution is determined.

### Cost Savings

In an effort to achieve the triple aims of the IHI, any opportunity to improve costs without sacrificing quality is beneficial. Preoperative education classes have shown a total cost savings averaging $4016 (27.2%) less than total costs for those patients who did not participate in preoperative education classes prior to elective hip or knee replacement [33]. While another study reported a cost savings of greater than $12,000 per year in those patients who attended an education course prior to surgery [24].

### Adult Education Techniques

Educating adult patients regarding surgical procedures can be accomplished by a variety of methods; in general, information tailored to the specific procedure in an interactive format provides the best results [95–97]. One key feature to improve learning and retention is through the use of repetition. Use of the *spaced retention method* increases memory retention up to 200% [98–100]. Spaced repetition is a learning technique that incorporates increasing intervals of time between subsequent reviews of previously learned material in order to exploit the psychological spacing effect [101]. Using the spaced retention method to repeat and reinforce patient expectations, goals, and the surgical information may be critical to improving outcomes. A comprehensive CP can provide spaced retention learning by aligning the surgeon, surgeon’s mid-level provider, surgeon’s office RN, Internal Medicine team, and the preoperative education instructors to teach correct detailed information in repetition at varying spaced intervals. We have accomplished this teaching method by reinforcing the goals and expectations for the patient’s surgery at each interaction with a healthcare provider leading up to surgery day (Table 1).

**Table 1:** Spaced retention method for preoperative education

	Provider	Visit	Goals
**1**	Surgeon–patient	Office	Discuss indications, expectations, goals, risks, and alternatives to surgery.
**2**	PA/APN/RN–patient	Office after MD	Reinforce the expectations and goals. Schedule surgery date. Give printed paperwork and joint replacement educational material.
**3**	IM/PCP–patient	Office <30 days prior to surgery	Reinforce the expectations and goals. Obtain preoperative labs and medically optimize.
**4**	Preoperative education class–patient and coach	Office <30 days prior to surgery, after IM/PCP visit	Reinforce the expectations and goals. Live demonstration with visual aids. Review printed educational material, tour facilities, arrange for any outpatient PT or DME needs. Identify any outstanding issues that may prevent home discharge.

### Interaction One

Patient education almost always starts in the office with the surgeon providing one-to-one education at the time when the decision for surgery is confirmed. This education session informs the patient of the expectations, risks, and alternatives to the upcoming surgery. In general, since surgeons have higher expectations than their patients, this office visit provides a great opportunity to clearly define *your* expectations for them regarding discharge, disposition, pain control, and overall satisfaction [42]. Although this one-on-one education may be the most important in gaining your patients trust, often very little of the medical details are retained and patients often leave the medical office with many unanswered questions after this visit.

### Interaction Two

Although not a separate visit, its importance cannot be understated. After the physician has discussed and answered questions regarding the risks, benefits, and alternatives of the surgery either a RN or mid-level provider sits with the patient to pick a surgery date, perform quick oral-dentition exam, and provide our hip and knee replacement print material. This is an opportune time to address any remaining questions, once again reinforcing our goals and expectations for their surgery.

### Interaction Three

A primary care physician (PCP) or internal medicine (IM) physician optimizes *all* patients prior to surgery. Uniformity of preoperative clearance and any preoperative instruction is critical to provide efficient, consistent, cost-effective preoperative evaluation. We recommend standardizing the preoperative workup, as well as the pre- and postoperative instructions as much as possible. This can be accomplished by using a team of physicians familiar with the expectations and goals of your surgery. We have found it most beneficial to use either the hospital employed medicine group or our own orthopedic Sports Medicine/PCP physician to perform all of our preoperative clearance consults. This allows for uniform preoperative workup, as well as reinforcement of our expectations and goals for their upcoming surgery.

### Interaction Four

The final interaction before surgery is the formal education class. It provides the best opportunity to discuss any remaining questions and recognize any unresolved concerns. The class is completed within 30 days of surgery and only after a PCP or IM physician has optimized or “cleared” the patient for surgery. A variety of teaching methods can be used for the education. In general, we use group sessions with live teaching from PowerPoint presentation and videos.

### Teaching Methods

One-to-one teaching is appropriate when sensitive or private topics need to be discussed; however, this process is time consuming and not cost-effective for large groups of patients [102]. Most commonly with elective hip and knee replacement patients, group teaching is most applicable and has been shown very effective [103, 104]. Other advantages to group teaching include the benefit of hearing answers to other questions from the other participants, group support, and modeling of behavior and skills by the group. Videotapes have been shown an effective way to educate as long as it delivers appropriate information in a short (less than 11 min) time period [105, 106]. Video education is even more effective when combined with live teaching from a healthcare provider [107]. Videotape and YouTube video teaching may be especially effective for low health literacy populations and those patients with high anxiety [82, 105, 108]. Fifty percent of patients responding to survey after attending a preoperative education prior to hip or knee replacement preferred verbal education, stating that this was clear and easy to understand [47]. While only 30% of respondents preferred videos or DVD’s, 10% found viewing of x-rays helpful and only 2% preferred written information [47].

### Education Class Structure

Required attendance for the education class should be *mandatory* prior to elective hip or knee replacement surgery. Strict compliance is critical to eliminate any confusion among surgeons and staff. When patients request to skip class or fail to attend, we cancel surgery until education class has been completed. Using slight modifications to the preoperative education guidelines recommended by Spalding et al., we suggest the following [18]:

- Avoid medical jargon
- Presenters should be the treating staff
- Structure programs chronologically
- Demonstrations should provide visual images and models
- Education classes should be taught on or near the joint replacement floor

Our classes are taught at the hospital where surgery will occur on the joint replacement floor. Allowing patients to familiarize themselves with the parking garage, hospital grounds, and even viewing the private rooms in which they will stay reduces anxiety about their upcoming surgery. The orthopedic joint replacement team members, including but not limited to the Physician Assistant, Advanced Practice Nurse, Physical Therapists, and Registered Nurses teach the education class.

All live slideshows and print content are approved by the joint replacement surgeons performing elective hip and knee replacement. After surgeon approval, all education material should be reviewed and edited for readability. Since low health literacy is associated with a poorer ability to understand and follow health instructions, poorer health outcomes, and poorer use of health care services, the readability of all patient education material is provided at a 4th grade reading level [109].

Our class sessions are taught in groups allowing time for discussion and questions. Incorporating group sessions and spousal support into the education program may facilitate an improvement in outcomes [28, 110]. For these reasons, our own educational program requires a “coach” be present during the education classes. In addition to the educational component of the class, our instructors also identify the patient’s social support system. We require patients to sign a non-binding contract stating they have read and understand the goals of our clinical pathway [8]. More specifically, they understand our goal is for discharge home post-operative day 1 after elective joint replacement. We require our patients identify in writing their “coach,” along with contact information for two additional family/friends that may be available on the day of discharge or in the immediate post-operative time period.

## Results of Our Clinical Pathway

Previously, we have shown that a well-coordinated CP with preoperative education as a key component can decrease LOS, minimize discharge to any facility other than home, lower readmission risk, and decrease cost [7•, 32]. More recently, we report a 14% reduction in costs per episode of joint replacement during participation in the CMS Bundled Payments for Care Improvement (BPCI) initiative [6]. In this study, we attribute the cost savings to maintaining a low LOS (2.13 days), high discharge to home rate (98.3% home), and low readmission rate. We believe we could not have achieved these results without a preoperative education class.

## Conclusion

Compelling evidence demonstrates the success of preoperative education classes for elective hip and knee replacement in the new landscape of bundled payment models. Despite the findings of the Cochrane Review study, we believe the data presented in this review article combined with our own experiences and data demonstrates preoperative education classes prior to elective joint replacement is a key component to successful participation in APM systems.
